# Prediction of precancerous cervical cancer lesions among women living with HIV on antiretroviral therapy in Uganda: a comparison of supervised machine learning algorithms

**DOI:** 10.1186/s12905-024-03232-7

**Published:** 2024-07-08

**Authors:** Florence Namalinzi, Kefas Rimamnuskeb Galadima, Robinah Nalwanga, Isaac Sekitoleko, Leon Fidele Ruganzu Uwimbabazi

**Affiliations:** 1 African Centre of Excellence in Data Science, University of Rwanda, PO BOX 4285, KK 737 St, Gikondo, Kigali, Rwanda; 2 College of Science and Technology, University of Rwanda, PO BOX 3900, KN 67 Street, Nyarugenge, Kigali, Rwanda; 3 London School of Hygiene & Tropical Medicine, London, England

**Keywords:** Cervical cancer, Supervised machine learning, Women living with HIV

## Abstract

**Background:**

Cervical cancer (CC) is among the most prevalent cancer types among women with the highest prevalence in low- and middle-income countries (LMICs). It is a curable disease if detected early. Machine learning (ML) techniques can aid in early detection and prediction thus reducing screening and treatment costs. This study focused on women living with HIV (WLHIV) in Uganda. Its aim was to identify the best predictors of CC and the supervised ML model that best predicts CC among WLHIV.

**Methods:**

Secondary data that included 3025 women from three health facilities in central Uganda was used. A multivariate binary logistic regression and recursive feature elimination with random forest (RFERF) were used to identify the best predictors. Five models; logistic regression (LR), random forest (RF), K-Nearest neighbor (KNN), support vector machine (SVM), and multi-layer perceptron (MLP) were applied to identify the out-performer. The confusion matrix and the area under the receiver operating characteristic curve (AUC/ROC) were used to evaluate the models.

**Results:**

The results revealed that duration on antiretroviral therapy (ART), WHO clinical stage, TPT status, Viral load status, and family planning were commonly selected by the two techniques and thus highly significant in CC prediction. The RF from the RFERF-selected features outperformed other models with the highest scores of 90% accuracy and 0.901 AUC.

**Conclusion:**

Early identification of CC and knowledge of the risk factors could help control the disease. The RF outperformed other models applied regardless of the selection technique used. Future research can be expanded to include ART-naïve women in predicting CC.

## Introduction

Cervical cancer (CC) is the fourth most common type of cancer among women with roughly 342,000 fatalities and 604,000 new cases worldwide in 2020 [1, 2]. Due to the absence of effective screening and Human Papillomavirus (HPV) vaccination programs, the majority of these new cases and fatalities occurred in low and middle-income countries (LMICs) [2]. Eastern Africa has the highest number of CC cases and deaths, with Malawi having the world’s highest age-standardized incidence and mortality rates of 40.1 and 28.6 per 100,000 respectively [2]. Uganda is the second-highest-incidence country in East Africa, with a CC incidence rate of 28.8 per 100,000 people annually, 6413 new cases, and 4301 deaths, placing it among the top ten countries worldwide [3]. Over 99% of CC cases are caused by Human Papillomavirus (HPV), and the primary mode of transmission between individuals is through sexual intercourse [4]. This makes at least half of the sexually active people have the HPV virus at some point in life though few will get cervical cancer [5]. However, there are other risk factors for CC including Sexually transmittable diseases (STDs) (like HIV, Chlamydia), multiple sexual partners, smoking, use of oral contraceptives, viral load status for WLHIV among others [2, 6, 7].Women Living with Human Immuno-deficiency Virus (WLHIV) have a six-folder increased risk of contracting CC as compared to their counterparts living without HIV and 5% of all the new cases diagnosed in 2018 were WLHIV [8]. However, those on Antiretroviral Therapy (ART) have a lower prevalence of high-risk HPV as compared to those who were ART naïve [9].

The WHO’s global strategy for eradicating cervical cancer seeks to attain a 90% HPV vaccination rate for girls by the age of 15, 70% of women being screened for the disease using high-performance tests by the ages of 35 and 45 years, and 90% of those who are diagnosed with the disease receiving treatment [10]. The secondary prevention measures include the screening of women for cancer lesions; this recommended the screening to start from the age of 30 years for women without HIV and 25 years for WLHIV as they are more at risk than the former [1]. With the high risk of HPV in WLHIV, the WHO first called for action to eliminate CC in 2018. The member countries were advised to have the mandatory screening of cancer lesions using various high-performance tests including HPV deoxyribonucleic acid (HPV DNA) that is highly recommended, visual inspection with Acetic Acid (VIA) which is commonly used in LMICs, and Conventional Pap Smear among others to increase the early detection of the disease [10]. All WLHIV on ART in Uganda between the ages of 25 and 49 years are advised to undergo CC screening, which is primarily conducted through VIA and those who screen positive with eligible precancerous lesions are treated by cryotherapy [11, 12]. However, the screening and vaccination against HPV are still low in LMICs [13].

Machine learning (ML) techniques in healthcare can aid in the early diagnosis of CC and precancerous lesions by leveraging the available data [14] which could reduce the costs involved in the screening. Various ML models have been applied to predict disease outcomes including Logistic Regression (LR), Decision trees (DT), Random Forests (RF), K-Nearest Neighbors (KNN), Support Vector machines (SVM) among other techniques [14–18]. However, these techniques have not been popularly used in predicting disease outcomes within Sub-Saharan Africa including Uganda. Furthermore, the applied models have focused on the general population of women with CC with few or no studies focusing on particularly those living with HIV. We therefore propose to assess the performance of these models in predicting CC among WLHIV on ART and identify some of the best predictors.

## Methods

The study used secondary data of 3025 women obtained from three health facilities in central Uganda at the level of Health Center IV (HC IV) that is Kajjansi HC IV, Ndejje HC IV and Kasangati HC IV. This is because HIV is more prevalent in this region [19]. It included all WLHIV who had been screened at least once for cervical cancer regardless of their ART start date. The facility in-charges were contacted to request permission to access the data, and the letter of acceptance to collect the data was signed.

### Variables of interest

The outcome variable, CC screening was categorized as “evidence of malignancy” for those who screened positive for CC and “no evidence of malignancy” for those who screened negative at facility level. Those who screened positive were coded as “1” and those who screened negative for CC were coded as “0” in the study.

The study included 16 variables of socio-demographic characteristics and clinical factors of 3025 WLHIV that had ever been screened for CC in the selected facilities. The selected demographic variables included age in years, occupation, body weight in kilograms and height in centimeters. The clinical factors included duration on ART in years, current ARV regimen coded as 1st line and 2nd line regimen, the method of family planning (FP) used coded as “No FP, hormonal and non-hormonal”, tuberculosis (TB) status coded as “No signs, TB suspect and on treatment”, ARV adherence as “good, fair and poor”, WHO HIV clinical stages 1, 2, 3 and 4, nutrition assessment as “normal, moderate acute malnutrition (MAM) and severe acute malnutrition (SAM)”, TB Preventive Therapy (TPT) status coded as “completed treatment, on treatment, never on TPT and stopped/removed”, advanced HIV disease status coded as “no advanced disease, suspected advanced disease and confirmed advanced disease”, Baseline CD4 count, CD4 count (current) and Viral load status as “detected and not detected”. Women who had viral load copies < 1000 were considered as not detected. These variables were selected based on the literature reviewed and data availability.

### Statistical methods

#### Data analysis

**Fig. 1:**
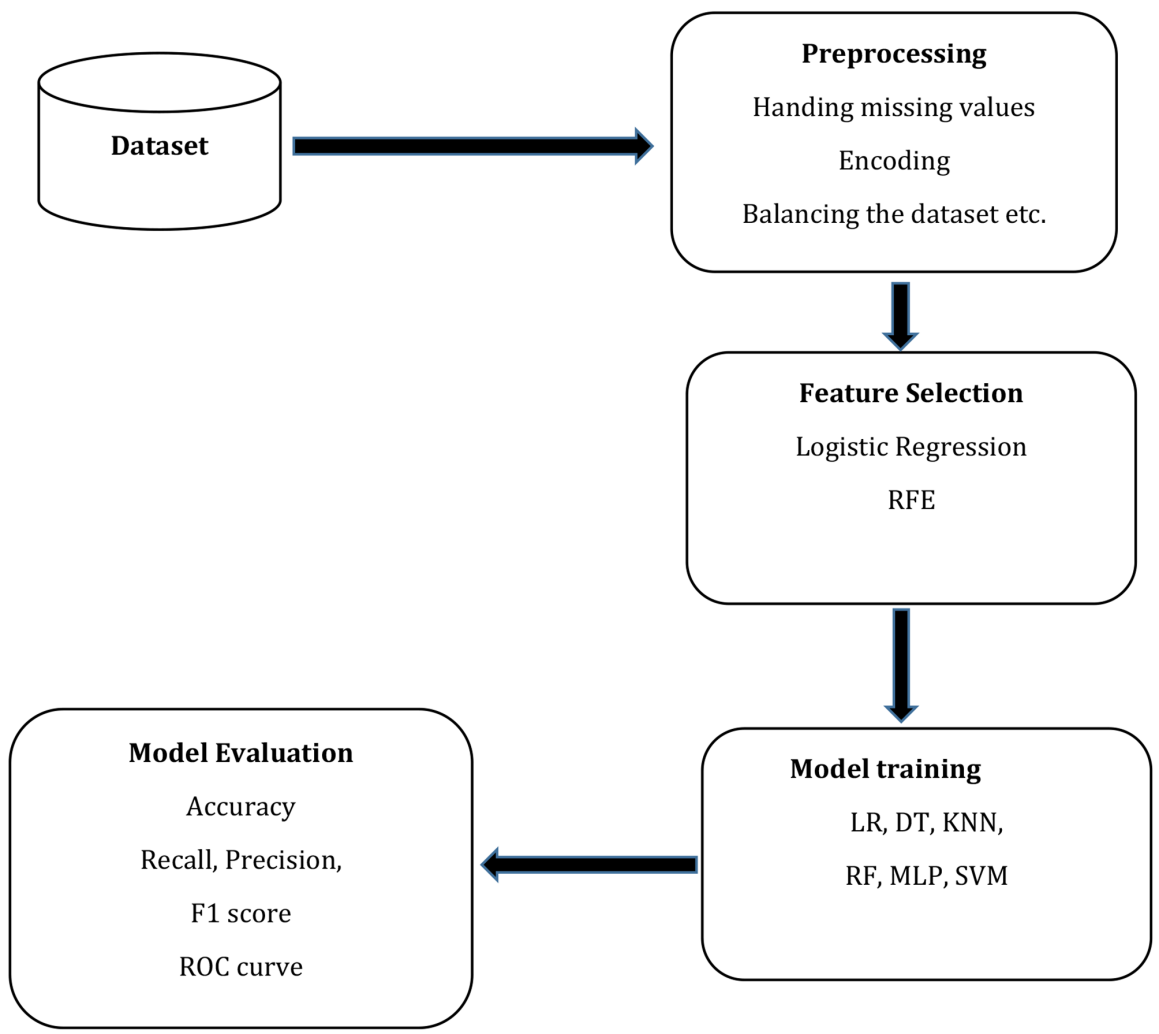
The workflow of the proposed methods

The Python programming software version 3.12 was used throughout the data analysis in this study. Data preprocessing involved several activities such as data cleaning to remove the noise from the data, handling missing data, outliers, transformation, and balancing classes among others depending on the nature of the data [17]. The KNN imputer was used to fill in missing values for qualitative variables and the median was used for quantitative variables as their data was highly skewed [20]. Furthermore, combining the Tomek Link resampling technology with synthetic minority oversampling (SMOTETomek) was used to balance the classes of those suspected to have CC and those without. This technique in the Python *imblearn* package uses both over-sampling and under-sampling to balance the classes and increase the model accuracy. To increase the minority class occurrences, SMOTE in SMOTETomek oversamples the minority class, and Tomek under samples the majority class to reduce noise while maintaining balanced distributions [21]. The Tomek links are pairs of the nearest neighbors of two classes that are close to each other. Using these links, the overlapping samples that SMOTE adds are removed [22]. The standard Scaler was used to standardize the numerical data. Figure 1 represents the flow chart of the proposed methods used in the study from data collection to the evaluation of the models.

### Machine learning techniques

As this is a classification problem, five supervised ML techniques were selected to be used in the prediction of CC in this study. These models were trained on the two sets of features as selected. These algorithms included;

#### Logistic regression (LR)

LR is one of the most used models for binary outcomes in Epidemiology. It’s a ML classification technique borrowed from statistics [14]. It’s commonly used when the outcome variable has binary outcomes for example yes/no, diseased/not diseased among others. LR does not assume a straight line connecting the explained and explanatory factors, but it shows how the output and predicted values relate to one another. Using the sigmoid or the logit function, the LR curve confines the results to 0 and 1. Like linear regression but uses the natural logarithm of the odds for the target variables instead of probabilities to build curves. Predictors don’t necessarily need to follow a normal distribution or have an equal variance across all groups [23].

#### Random Forest (RF)

A forest-like structure made up of many decision trees makes up the classification approach and ensemble method known as RF. The bagging approach is another name for it, and it may be applied to classification and regression (CART) problems. [14]. DTs are generated randomly from the training set’s partial set using the information gain or the GINI index. Having more trees increases stability. The features categorization and target variable are built individually from each DT as the tree casts a vote for that class. The RF then selects the classification with the most votes if there is a classification challenge, or if there is a regression challenge, it determines the mean of all the trees. [23].

#### K-Nearest Neighbors (KNN)

KNN is a lazy-learner and easy-to-implement supervised Machine Learning Algorithm. It has multiple uses, that is, can be used as both a classification and regression as well as handling the missing values in a dataset and resampling. It classifies a new data point based on the k-neighbors as its name states to get its class [14]. It calculates the Euclidean distance of the neighboring points and sees which class label is much closer to the new unknown data point. The class label with k neighbors that are very close to the unknown point (with the shortest Euclidean distance) wins the new point. The k is a pre-determined number of neighbors that are initially randomly selected and it’s updated until the model achieves the best accuracy.

#### Support Vector Machine (SVM)

Identifying a hyperplane that maximizes the margin between two specified classes while reducing the penalty factor is the primary objective of SVM [14, 16]. If the data can be separated linearly, the linear SVM is employed; otherwise, kennel trick approaches are used. A key element of an SVM that converts lower-dimensional data into higher-dimensional space and can distinguish between various classes is its kernel. Kennel tricks convert the classes into forms that can be linearly separated before fitting the SVM model. The Radial Basis Function (RBF), sigmoid function, and the polynomial function are some of these Kennel Tricks. If the classes are originally inseparable, all of these strategies can be used to choose the optimal model.

#### Multi-Layer Perceptron Neural Network (MLP-NN)

A neural network is a machine learning technique that mimics the brain of a human being using neurons. It consists of different layers, that is, the input layer that consists of the number of inputs/features. MLP has weights that are initially randomly selected and later on keep on updating back to front until the model best performs [16, 24]. It also consists of hidden layers; this helps to hyper-tune the model to perform better. It also consists of the output layer with the number of neurons corresponding to the classes of the target variable. Different optimizers were implemented to determine which set best performs. These optimizers include the sigmoid, relu, SDG, and Adams.

### Model evaluation

The trained models were evaluated using unseen data from the testing set to determine its efficacy. In the medical field, the datasets are highly unbalanced, that is the proportion of those with the disease are far less than those without the disease, therefore, we can not only rely on accuracy to evaluate the model as it may be biased toward the majority class [14, 23]. In this study, the confusion matrix, and the Receiver Operating Characteristic (ROC) curve were used to evaluate the models. The True Positives (TP), False Negatives (FN), True Negatives (TN), and False Positives (FP) variables make up the confusion matrix. TP shows the diseased that where correctly predicted as being with the disease. FN show the diseased that were wrongly predicted as not diseased. The aim is to always reduce the FN as much as possible. FP indicate those that are not diseased but wrongly predicted as diseased. TN indicates those that were not diseased and are correctly predicted as having no disease. The accuracy, recall, precision and F1_score of the model can be calculated from the confusion matrix.

**Accuracy** is the most common evaluation metric that is used. It identifies all the TP and TN that were predicted by the model. It is calculated as below.

$$\text{A}\text{c}\text{c}\text{u}\text{r}\text{a}\text{c}\text{y}=\frac{\text{T}\text{P}+\text{T}\text{N}}{\text{T}\text{P}+\text{T}\text{N}+\text{F}\text{P}+\text{F}\text{N}}$$

**Recall** is also known as sensitivity if relating to TP or specificity if relating to TN. It’s mostly used to refer to sensitivity as the aim is always to identify the those with the disease.

- **Sensitivity** gives the percentage of the true positives that were correctly predicted by the model out of the total/actual positives. It’s calculated as below.

$$\text{S}\text{e}\text{n}\text{s}\text{i}\text{t}\text{i}\text{v}\text{i}\text{t}\text{y}=\frac{\text{T}\text{P}}{\text{T}\text{P}+\text{F}\text{N}}$$

**Precision** is also known as Predictive Accuracy (PA). It looks at the columns of the predicted values and identifies which values were predicted correctly out of all the predictions. It can be;

- **Positive Predictive Accuracy (PPA)** gives the proportion of the TP out of all those that were predicted as having the disease. PPA is commonly referred to as precision. It is calculated as shown below.

$$\text{P}\text{P}\text{A}=\frac{\text{T}\text{P}}{\text{T}\text{P}+\text{F}\text{P}}$$

**F1 score** calculates the harmonic mean of precision and recall and compared to the accuracy measure, it provides a more precise assessment of the number of misclassification instances [17]. It is mathematically computed as below.

$$\text{f}1\ \text{s}\text{c}\text{o}\text{r}\text{e}=2\text{*}\frac{\text{P}\text{r}\text{e}\text{c}\text{i}\text{s}\text{i}\text{o}\text{n}\text{*}\text{r}\text{e}\text{c}\text{a}\text{l}\text{l}}{\text{P}\text{r}\text{e}\text{c}\text{i}\text{s}\text{i}\text{o}\text{n}+\text{r}\text{e}\text{c}\text{a}\text{l}\text{l}}$$

**ROC Curve**

The Area under the ROC is a metric that is used in the classification of binary problems. The sensitivity (TP Rate) and specificity (FP Rate) at various thresholds are plotted on this graph. It is among the most widely used evaluation metrics, especially in the health sector. The likelihood that a classifier will rank a randomly selected positive element higher than a randomly selected negative element is known as the Area Under the Curve (AUC) of the classifier [25]. An independent distinction between the positive and negative classes can be made by the model/classifier when the AUC is one. Indicating that the model will be able to recognize more true values (TP and TN) than the FP and FN, the AUC should be between 0.5 and 1. If the AUC is less than 0.5, the model is not good since it cannot distinguish between the positive and negative classes, and if the AUC is more than 0, the model will classify all of the points as negatives.

## Results

The results in Table 1 for the multivariate binary LR revealed that six features were related to CC. most of these features (duration on ART, Viral load status, current ARV regimen and WHO HIV clinical stage 4) were protective factors as their Adjusted Odds Ratios (AOR) were < 1 and only the TPT status was a risk factor as its AOR was > 1. The findings indicated that initiating ART and retaining its uptake is very crucial on the CC screening of a woman as an additional increase in duration on ART decreases the odds of screening positive for CC lesions by 0.96 times (AOR: 0.96 95%CI: 0.94, 0.98). Family planning type used was also important in the screening: those women who used non-hormonal and those who did not use FP at all were 0.17 times and 0.68 times less likely to screen positive for CC as compared to those that used hormonal FP methods times (AOR: 0.17 95%CI: 0.112, 0.23) and (AOR: 0.68 95%CI: 0.60, 0.77) respectively. The viral load status is very important for any person under ART care and treatment as it tells whether one is suppressing the virus or not. The results showed that those women that were suppressing (viral load not detected) were 0.44 times less likely to screen positive for CC compared those their non-suppressing counterparts (AOR: 0.44 95%CI: 0.38, 0.50). TPT is a therapy used to prevent those living with HIV from contracting TB disease. The results indicated that those patients that were currently on TPT treatment and those that had stopped/removed from treatment due to side effects were 2 times and 10 times more likely to screen positive for CC than those who were never initiated (AOR: 2.02 95%CI: 1.39, 2.97), (AOR: 9.79 95%CI: 5.67, 16.92) respectively. These significant features were later considered for CC prediction. Some variables such as age and BMI were not considered at multivariate level due to multicollinearity issues.

**Table 1:** The multivariate binary logistic regression analysis of the risk factors/features for CC

Variable	Multivariate analysis	
	AOR (95%CI)	P_Value
**Duration on ARV**	0.96 (0.943, 0.976)	**< 0.001**
**Current ARV Regimen**		
1st line	1*	
2nd line	0.027 (0.006, 0.111)	**< 0.001**
**Family Planning method**		
Hormonal	1*	
Non-hormonal	0.166 (0.119, 0.230)	**< 0.001**
No FP	0.684 (0.603, 0.777)	**< 0.001**
**WHO HIV clinical stage**		
Stage 1	1*	
Stage 2	0.882 (0.580, 1.341)	0.557
Stage 3	1.123 (0.574, 2.194)	0.735
Stage 4	0.149 (0.030, 0.730)	**< 0.05**
**TPT Status**		
Never	1*	
On treatment	2.017 (1.387, 2.965)	**< 0.001**
Removed/stopped	9.792 (5.667, 16.920)	**< 0.001**
Completed treatment	1.26 (0.891, 1.781)	0.192
**Viral load status**		
Detected	1*	
Not detected	0.439 (0.383, 0.502)	**< 0.001**

1* represents a reference category

**Fig. 2:**
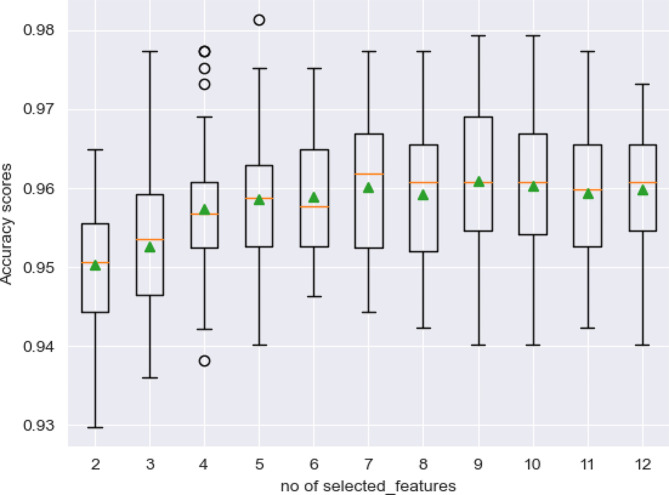
Box and whisker plot showing the number of features selected by RFE

The findings in Fig. 2 show the box and whisker plot that visualized the distribution of the accuracy scores versus the number of features selected. It can be observed that the accuracy score increased with the increased number of selected features. The peak was obtained when the number of features selected was 7 with an accuracy of approximately 96% after which it fluctuated. The seven selected features included age, BMI, ART duration, family planning, WHO HIV clinical stage, TPT status, and Viral load status. These were also later used in the prediction of CC.

It can be observed that four variables that is; duration on ART, WHO clinical stage, TPT status, Viral load status, and family planning were selected by both techniques that were deployed. This implies that they are very important factors that should be considered in the CC screening of WLHIV.

The results in Tables 2 and 3 represent the models performance using both the LR-selected and RFE-selected features respectively. In Table 2, the results indicated that the RF outperformed the other models in most of the metrics with scores of 91%, 79%, 85%, 86% for precision, recall, F1-score and accuracy respectively with SVM and LR being the worst performers. In Table 3, the results revealed that RF, MLP, and KNN were the best-performing models here with accuracies of 90%, 88%, and 86% respectively. SVM was also good with an accuracy of 75% and the binary LR model had the worst performance of all with an accuracy of 61%.

**Table 2:** Evaluation of models from logistic regression selected features

Algorithm	Precision (%)	Recall (%)	F1_score (%)	Accuracy (%)	AUC
KNN	64	88	74	69	0.695
SVM	70	52	60	66	0.653
LR	56	58	57	57	0.568
RF	91	79	85	86	0.857
MLP	68	66	67	68	0.676

**Table 3:** Evaluation of models from RFE-selected features

Algorithm	Precision (%)	Recall (%)	F1_score (%)	Accuracy (%)	AUC
KNN	81	95	87	86	0.864
SVM	73	77	75	75	0.749
LR	60	64	62	61	0.613
RF	91	88	90	90	0.901
MLP	85	92	89	88	0.885

**Fig. 3:**
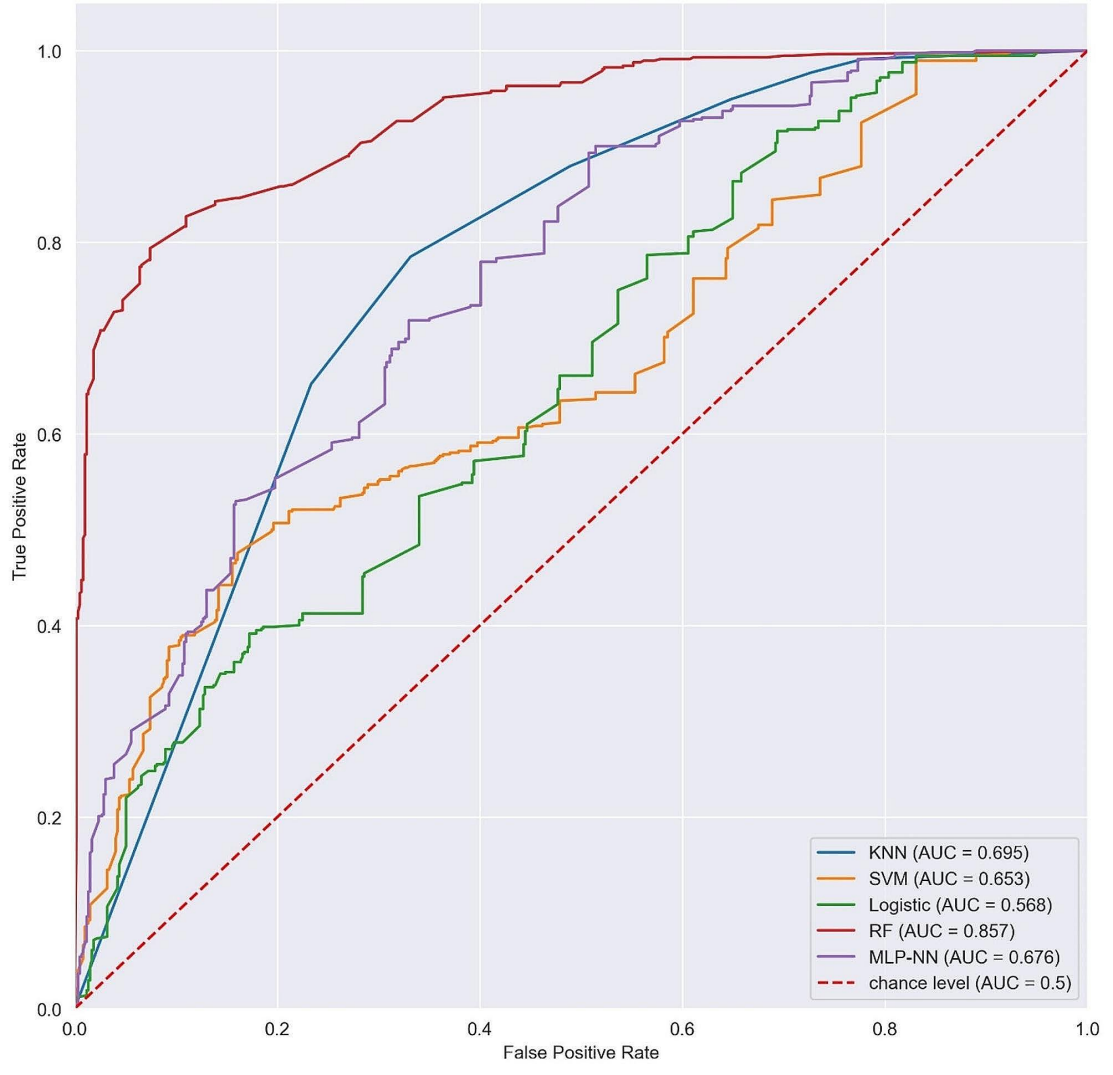
ROC curves showing performance of the models from LR-selected features

**Fig. 4:**
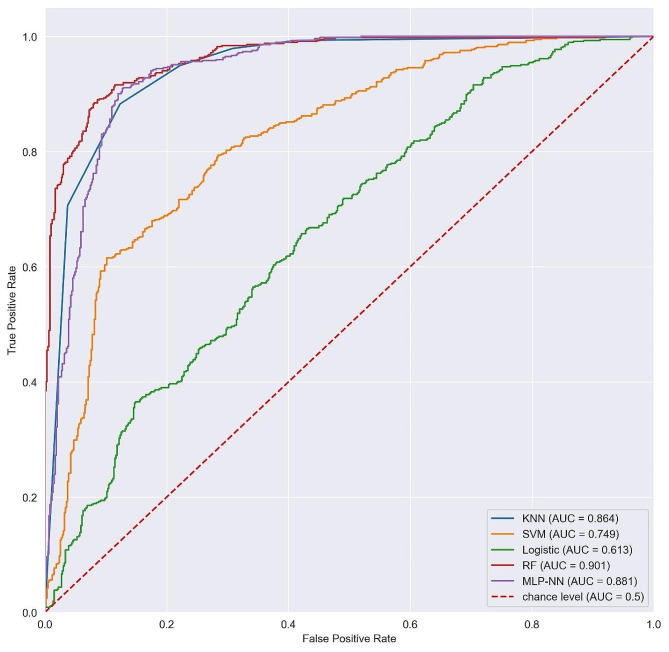
ROC curves showing the performance of the models from RFE-selected features

Considering the ROC, Fig. 3 shows the performance of models from LR-selected features. The findings show that RF was far better than the other models with AUC of 0.857. Similarly, the findings in Fig. 4 for models from RFE-selected features revealed that RF with AUC of 0.901 outperformed here and its AUC was better than that for LR-selected models. The MLP-NN also performed well from RFE with an AUC of 0.885, that is above that of all the models from LR-selected features. These models showed they can better predict CC than any other of the considered models.

## Discussion

This study intended to identify the supervised ML algorithm that best predicts and predictors of CC in the WLHIV in Uganda. The duration on ART, WHO clinical stage, TPT status, Viral load status, and family planning method used were selected by both techniques that were deployed for feature selection. This implies that these four features are very crucial in the prediction of CC. Our findings suggest that RF from RFERF-selected features was the best predictive model for CC with an accuracy of 90% and AUC of 0.901. This is in line with [17, 26] that had RF as one of their best performing models in CC prediction.

Based on our results from LR, TPT status was highly associated with CC as compared to other features. Those who were stopped from treatment due to side effects were 9 times more likely to screen positive for CC compared to those who had never been initiated on TPT. This may need further research to know the reason behind it as it is not having much literature written on it currently in relation to CC. TPT has however shown to be a cost-effective way to lower TB incidence, morbidity, and mortality among persons living HIV (adults and children) [27, 28]. The duration on ART is also of significant importance for CC screening and positivity screening decreases with the increasing duration on ART. These results are in agreement with the study by [9] which revealed that women on ART have a reduced risk/ low prevalence (AOR = 0·83, 95% CI 0·70–0·99) of high-HPV as compared to those not on ART and this was adjusted for CD4 count and the duration they had spent on ART. Our results were also further supported by [29] that concluded that those WLHIV in resource limited settings who were not on ART were 2.21 times (AOR = 2.21, 95% CI (1.28–3.83)) more likely to have CC lesions than those on ART. We also found out that age is one of the important predictors of CC screening outcomes by RFERF. Our results were correlating with those from a study carried out in Rwanda by [30] that concluded that the HPV infection decreased (0.52 times) by the age of a person. This may further be attributed to the fact that older women tend to be less sexually active than the young ones as it was seen that HPV is mostly spread through sexual intercourse [4, 31]. Our findings also partially correlated with a study by [6] in Nigeria, their study found out that positivity screening decreased with an increase in age, with women at least 40 years having a lower relative risk (RR = 0.4; 95%CI = 0.2–0.7). However, our study contradicted this study as the positivity screening for CC was related to Contraceptive (Family Planning) use, WHO clinical stage, and current ART regimen yet they were seen not to have any relationship with CC in their study. This contradiction may be because in our study, only WLHIV on ART were included in the study as opposed to the other one that also included those that were ART naïve thus difference in the study populations considered. In contrast to other studies, our study shown that CD4 count was not a good predictor of CC among WLHIV [29, 32]. This difference may be attributed to the variation in the study populations considered.

Various ML models have been applied in various studies to predict CC in different countries [15–18]. However, there is limited literature focusing on the WLHIV and thus originality of our study. After the application of the selected ML models, RF outperformed the rest of the models regardless of the feature selection technique used. Our study results are similar and correlated to several studies that identified RF as one of the best model in the prediction of cervical cancer. Our results were similar to a study by [17] that predicted CC using ML algorithms and concluded that RF, DT, Adaptive, and Gradient boosting that each at an accuracy of 100% were the best predictors of CC. Our models are also further supported by the study by [26] that used supervised ML algorithms to classify CC that concluded that DT with RFE and SMOTETomek had the accuracy and sensitivity/recall of 98.72% and 100% was a good model for the classification of CC. However, a study by [25] had the KNN shining above the DT and RF with its AUC of 0.822 as compared to 0.52 and 0.532 of DT and RF respectively. But compared to our study, the AUC of KNN (0.822) in their study was less than what was achieved for RF in this study which makes our findings more superior. With several studies having RF performing better in predicting CC regardless of which study population considered, this indicates that this models can be trusted in the proper classification of CC as supported by our latest prediction among WLHIV in Uganda. However, some other studies proposed new models that performed well in the classification of CC. Furthermore, models that did not perform well in our study were shining in some studies. A study by [18] proposed a model that worked on a deep learning model that was supported by XGBoost that yielded an accuracy of 96.5% compared to the models that existed. Also, a study by [15] that also used supervised ML algorithms to predict CC concluded that the QUEST and C&R trees outperformed other models in the prediction with accuracy, sensitivity, specificity, and AUC of (95.55%, 90.48%, 100%, 95.20%) and (95.55%, 90.48%, 100%, 95.20%) respectively. Furthermore, a comparative analysis study by [24] found that SVM and LR had the best scores of Precision, Recall, F1 Score, and Accuracy and thus recommended their use in the classification and prediction of CC yet in our study, SVM and LR have been seen trailing throughout our modeling process. This may be due to the difference in the study populations and the techniques used in the selection of features.

## Conclusion

The likelihood of effective treatment throughout the pre-cancer and cancer stages increases with early detection, and being alert to any signs and symptoms of cervical cancer can help prevent diagnostic lags. Some of the most important predictors of CC in WLHIV in Uganda that were identified in this study included duration of ART, the viral load status, method of family planning, TPT status among others. More accurate disease prediction is now achievable thanks to machine learning. As proved by this study, the RF model from RFERF selected features suggested in this work can be utilized to predict CC among WLHIV. However, CC screening is still low in Uganda and thus there is a need for policy makers to come up with measures to improve.

Future research can be done with the inclusion of ART naïve women in the study and try other ML that this study has not applied. Additionally, more work can be done on the comparison of feature selection using the traditional methods of testing for significance and the use of ML techniques to observe whether the same features are selected by these techniques.

## Data Availability

The datasets used and/or analysed during the current study available from the corresponding author on reasonable request.

## Abbreviations

CC: Cervical Cancer
DT: Decision Tree
HIV: Human Immuno-Deficiency Virus
HPV: Human Papillomavirus
KNN: K-Nearest Neighbor
LR: Logistic Regression
ML: Machine Learning
MOH: Ministry of Health
MLP: Multi-Layer Perceptron
RF: Random Forest
RFE: Recursive Feature Elimination, RFERF: Recursive Feature Elimination with Random Forest
SVM: Support Vector Machine
WHO: World Health Organization
WLHIV: Women Living with Human Immuno-Deficiency Virus
